# Mixed Ionic and Electronic Conduction in TeO_2_-ZnO-V_2_O_5_ Glasses towards Good Dielectric Features

**DOI:** 10.3390/ma15217659

**Published:** 2022-10-31

**Authors:** Imen Mechrgui, Amira Ben Gouider Trabelsi, Fatemah. H. Alkallas, Saber Nasri, Habib Elhouichet

**Affiliations:** 1 Sciences Faculty of Tunis, University of Tunis El Manar, Tunis 2092, Tunisia; 2 Department of Physics, College of Science, Princess Nourah bint Abdulrahman University, P.O. Box 84428, Riyadh 11671, Saudi Arabia; 3 Laboratory of Spectroscopic Characterization and Optic Materials, Faculty of Sciences, University of Sfax, B.P.1171, Sfax 3000, Tunisia; 4 Physics Department, College of Sciences, University of Bisha, P.O. Box 551, Bisha 61922, Saudi Arabia; 5 Laboratory of Characterizations, Applications and Modelisation of Materials LR18ES08, Sciences Faculty of Tunis, University of Tunis El Manar, Tunis 2092, Tunisia

**Keywords:** tellurite glasses, ionic conduction, polaronic hopping, high dielectric constant, dielectric loss, modulus

## Abstract

The melt-quenching technique was used to synthesize tellurite glasses of the chemical composition 80TeO_2_-(20-x) ZnO-xV_2_O_5_. X-ray diffraction (XRD) patterns indicate the amorphous nature of the prepared glasses. Raman and FTIR measurements demonstrate a progressive substitution of the Te-O-Te linkages by the Te-O-V bridges and the formation of VO_4_ and VO_5_ units by a change of the vanadium coordination due to the higher number of oxygens incorporated by further addition of V_2_O_5_. The AC conductivity was investigated in the frequency range of 40 Hz to 10^7^ Hz between 473 K to 573 K. A good coherence of the AC conductivity was found using a model correlating the barrier hopping (CPH) and the dominant conduction process changes from ionic to polaronic with the addition of V_2_O_5_. The dielectric constant exhibits high values in the range of lower and medium frequencies. Both variations of the electric modulus and the dielectric loss parameters with frequency and temperature showed a relaxation character mainly assigned to the vanadate phases. The electric modulus displays a non-Debye dielectric dispersion and a relaxation process. The present results open the door to future zinc-tellurite glasses-doped vanadium exploitation as a potential electrolyte-based material for solid-state batteries.

## 1. Introduction

Zinc-tellurite glass is extensively known to have good chemical durability and transmission capability, high dielectric constant and refractive indices, perfect infrared transmission, and low melting points. This kind of glass has been widely applied in industry in memory devices, micro-structured optical fibers, and switching due to its good semiconducting properties [1,2,3]. Recently, zinc-tellurite glass doped with heavy metal oxides has received great scientific interest due to the major role that such oxides play in improving optical and electrical properties of these glasses [4,5,6,7].

In earlier studies [8,9], we showed that structural, optical, electrical, and dielectric properties of glass are extremely dependent on the glass composition. Particularly, the addition of V_2_O_5_ to the glassy network highly contributes in reducing dielectric losses through the activation of the conduction mechanism by providing V^4+^ and V^5+^ ions in the valence state, which favors the hopping of small polaron [8,10]. Thus far, the structural, vibrational, electrical, dielectric, and modulus properties of tellurite glass doped using various amounts of V_2_O_5_ need further investigations and correlations between all the results to understand the key rules of vanadate ions in glass structure and its conduction process. This will identify the vanadate ions’ impact on the modification of the network structure and consequently will define the conduction mechanism and dielectric constant of the glass through V_2_O_5_. Such a find will advance energy storage and nonlinear optical applications.

To the best of our knowledge, only a few studies so far considered the effect of V_2_O_5_ on the structural, thermal, vibrational, electrical, and dielectric properties of zinc-tellurite glass. Herein, we investigate all these properties on (80-x)TeO_2_ + 20ZnO + xV_2_O_5_ glasses with various concentrations of V_2_O_5_ (x = 0, 5, 10, and 15 mol %). The electrical conductivity and dielectric studies cover a wide frequency region (40 Hz to 10^7^ Hz) and a wide temperature range varying from 423 K to 523 K. This elucidates the changes of the conduction mechanism in response to the variation of the frequency and V_2_O_5_ composition. Furthermore, the dependency of the dielectric constant and dielectric loss as a function of frequency, temperature, and V_2_O_5_ content are determined. The suitability of the present glass for devices application is provided.

## 2. Experimental

Vanadium-doped zin-tellurite glasses with the chemical composition (80-x) TeO_2_ + 20 ZnO + xV_2_O_5_ (x = 0, 5, 10, and 15 mol %) were prepared using the traditional melt-quenching method. The prepared samples are denoted as TZV0, TZV5, TZV10, and TZV15. Commercial reagents in the form of TeO_2_, ZnO, and V_2_O_5_ powders with 99.99% purity were well-mixed in appropriate proportions in a tungsten crucible and melted in an electric furnace at 900 °C for 2 h. To avoid the thermal chocs and release the remnant mechanical stress, we first rapidly quenched the melt by dropping into a stainless-steel plate maintained at 200 °C; then, immediately after quenching, the as-prepared samples were annealed at 300 °C (<Tg: glass transition temperature) for 2 h to be later placed at room temperature to cool down slowly. Therefore, the glass was polished using a fine emery paper to prepare disk-shape samples (of a diameter (d = 12 ± 0.1 mm) at a thickness (e = 2 ± 0.1 mm)) suitable for the optical and electrical characterization.

X-ray diffraction (XRD) patterns were recorded at room temperature using a Philips X’pert diffractometer (Philips, Amsterdam, The Netherland) equipped with Cu X-ray tube (λ = 1.54 A°), at 40 kV and 100 mA. For thermal measurements, differential scanning calorimetry (DSC) technique (Melter Toldo DSC823e) was used (Mettler Toledo AG, Switzerland). The DSC scans were performed on 20 mg glass powder at a heating rate of 10 ºC/min in N_2_ atmosphere using platinum pans. Raman spectra were measured using Labram HR spectrometer (Horiba Scientific, Irvine, CA, USA) and He-Ne laser (λ = 632 nm) as the excitation source. The structures of all the glass samples were examined via Perkin-Elmer (FTIR 2000, Waltham, MA, USA) spectrometer, and all IR transmittance spectra were collected in the range of 400–1200 cm^−1^ with a resolution of 4 cm^−1^. An Agilent 4294A (Santa Clara, CA, USA) impedance analyzer was utilized to measure the complex impedance versus the frequency (40 Hz–10^7^ Hz) at different temperatures (240 °C–360 °C).

## 3. Results and Analysis

### 3.1. DSC, XRD, Raman, and FTIR Analysis

**Figure 1:**
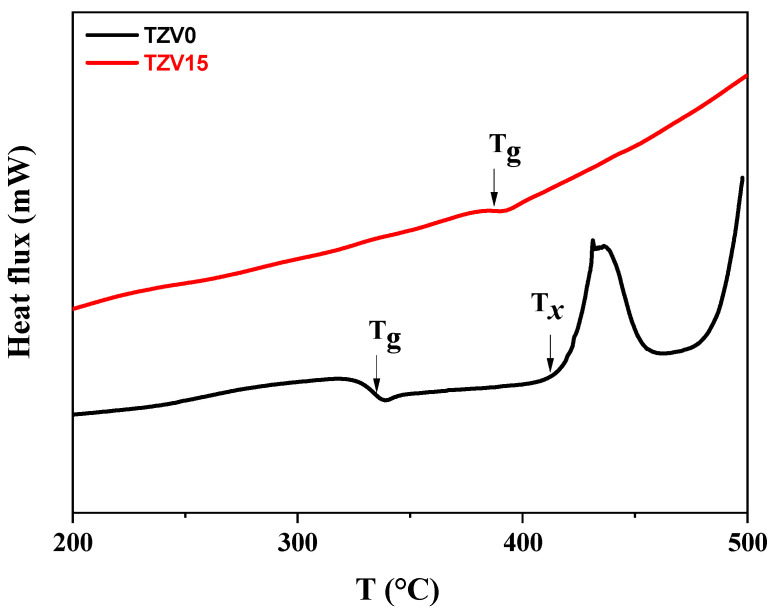
DSC curves of the as synthesized glasses.

From the DSC curves (Figure 1), the glass transition temperature Tg was estimated to 332 °C and 386 °C for the samples TZV0 and TZV15, respectively, with an accuracy of ±3 °C. The tendency of Tg is to increase with the added amount of V_2_O_5_, which contributes to the good thermal stability of the TZV glasses.

**Figure 2:**
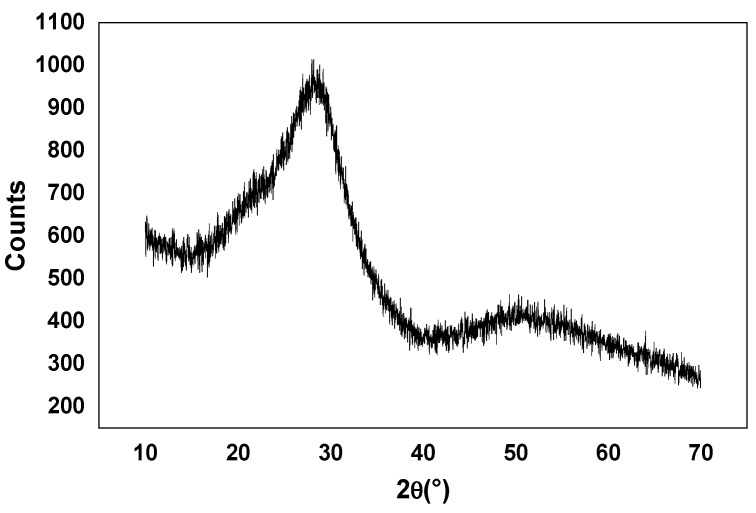
X-ray diffraction pattern of the TZV5 glass.

Figure 2 represents a typical XRD pattern relative to the TZV5 glass after thermal treatment at 300 °C. A broad scattering at lower angles was the only observed signature of disordered structure. This evidenced the amorphous nature of the prepared glasses.

**Figure 3:**
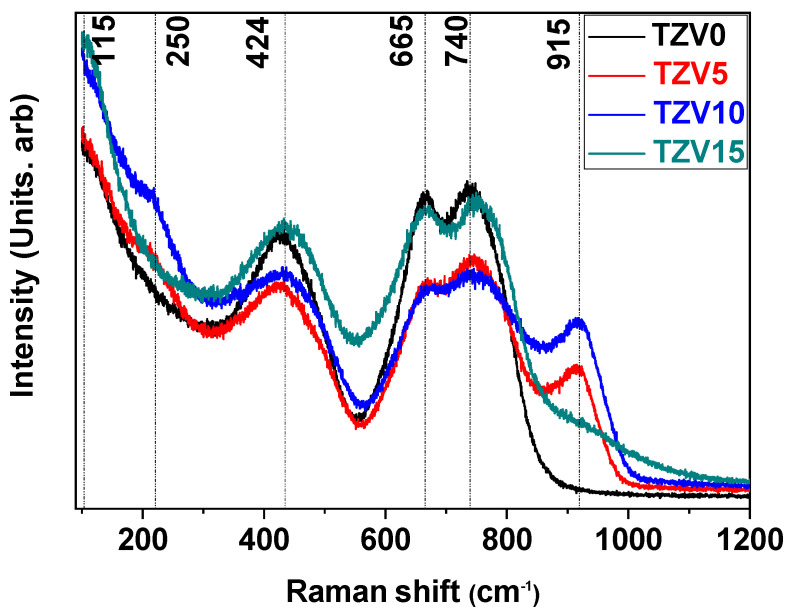
The Raman spectra of the TZV glasses.

The Raman spectra of the glasses are illustrated in Figure 3. The band appearing at low frequencies (around 115 cm^−1^) is relative to the Boson vibration. This latest development is defined as the collective motions of atoms’ acoustic phonons within the medium-range of glasses that characterize the disorder and the amorphous structures [6]. The Raman band at 210 cm^−1^ corresponds to the vibrations of trigonal pyramidal (tp) TeO_3_ groups overlapped with bands originated by V-O-V and/or V-O-Te vibrations [11]. On the other hand, the band at 424 cm^−1^ is ascribed to both the bending and stretching vibrations of Te-O in Te-O-Te or O-Te-O linkages, where oxygen is alternatively in an axial or equatorial position [5]. The large Raman band at 665 cm^−1^ is relative to Te-O stretching vibration in the (TeO_4_) tbp units [5,12]. Brovelli et al. [13] suggested that this band is relative to the crystalline phase α-TeO_2_. The shoulder appearing at about 740 cm^−1^ is reported to be due to the presence of non-bridging oxygen (NBO) in some tellurium structural units [5,14]. The band situated around 915 cm^−1^ is ascribed to the V-O stretching vibration in (VO_4_) units [15]. As the V_2_O_5_ content increases from 0 to 15 mol %, the Raman bands slightly shifted to a higher frequency, while their intensities decreased owing to the generation of NBOs at both the equatorial and axial positions, which leads to the formation of new bonds of Te-_eq_O-V^+^, Te-_ax_O-V^+^, and also Te=O.

The shoulder appearing at higher frequencies (1000 cm^−1^) for 15 mol % of V_2_O_5_ is explained from the contribution of the new vibrations V=O (relative to the (VO_5_) units) and V-NBO [16]. The spectral recovery between all the Vanadyl vibrations results in the observed shoulder. The presence of both (VO_4_) and (VO_5_) units evidences the existence of the V^4+^ and V^5+^ ions in the glassy network.

Thus, the evolution of vanadate vibrations underlines the destruction of Te-O-Te chains and the tendency of V_2_O_5_ to act as network former with creation of V^4+^, V^5+^ ions, and NBO defects.

**Figure 4:**
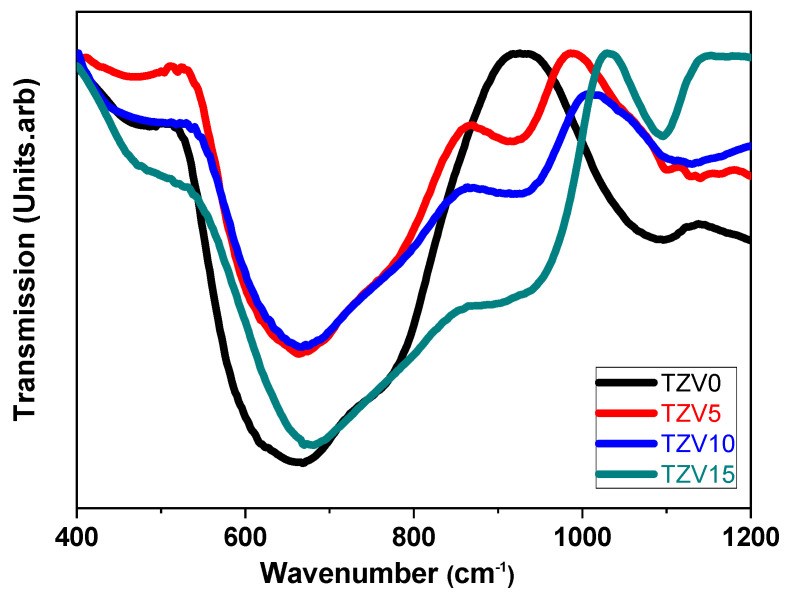
The FTIR spectra of the TZV glasses.

The FTIR spectrum of the TZV0 sample showed principally three peaks located at 470, 670, and 920 cm^−1^ (see Figure 4). The band located at around 470 cm^−1^ is assigned to the bending vibrations of Te-O-Te or O-Te-O linkages [5]. The broader band positioned between 600 and 750 cm^−1^ is an overlapping of two bands: the first, centered at 670 cm^−1^, is related to the stretching vibration of equatorial and axial Te-O bonds in the (TeO_4_) trigonal bi-pyramid units (bridging oxygen (BO)) [11]. The second band that appears above 700 cm^−1^ is ascribed to the Te-O vibration in (TeO_3_) trigonal pyramid units (tp) with non-bridging oxygen (NBO) [5]. At a lower amount of V_2_O_5_, the vanadate ions enter the glass network by breaking up the Te-O-Te, bonds resulting in dangling bonds (Te-O-V) that decrease TeO_3_ units by forming (TeO_4_) ones [14]. The variation of the intensity depends highly on the increase of the number of TeO_3_/TeO_3+1_ units at the expense of the number of (TeO_4_) units. The appearance of a new band centered at around 920 cm^−1^ is related to the stretching vibrations of V=O linkages in the pentahedral (VO_5_) units [15].

In conclusion, both Raman and FTIR analyses revealed a partial transformation of VO_4_ units to VO_5_ by a change of the vanadium coordination due to the higher number of oxygens incorporated by the addition of V_2_O_5_.

### 3.2. Impedance Spectroscopy

#### 3.2.1. Nyquist Spectra

**Figure 5:**
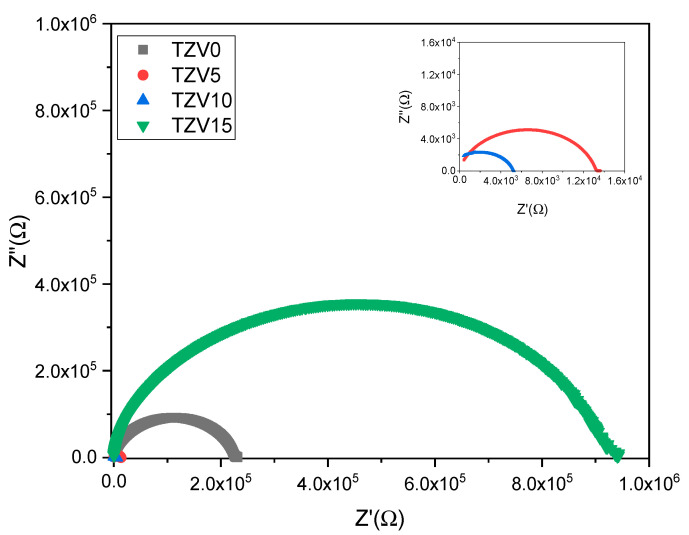
Cole–Cole plot of the TZV glass samples at 340 °C.

Figure 5 shows a complex impedance Cole–Cole plots for the different glass samples, i.e., TZV0, TZV5, TZV10, and TZV15, at the temperature T = 340 °C. The inset figure represents the suggested equivalent circuit to investigate the electrical properties of the glasses. In these semi-circles, a slight degree of decentralization can be detected since their centers are located below the axis of the real part *Z*′ of the impedance. The result shows a non-Debye-type relaxation for all samples. The deviation to a Debye profile in the current system could be attributed to the formation of macroscopic dipole groups and/or the formation of non-polar clusters [17].

In this regard, we may consider each semicircle equal to a circuit composed of two parallel RC elements connected in series. As the semicircle is dissymmetric, and its center is below the real axis, we used a constant phase element (CPE) instead of the ideal capacitor. The total impedance of the equivalent circuit is given by:

(1) $$Z*=Z\prime +jZ\prime \prime ={\left(\frac{1}{R_{b}}+\frac{1}{Z_{CPE_{b}}}\right)}^{-1}+{\left(\frac{1}{R_{int}}+\frac{1}{Z_{CPE_{int}}}\right)}^{-1}$$

where *Z*′ and *Z*″ designed the real and imaginary parts of the total impedance.

The impedance of the *CPE* is defined via [18]:

(2) $$Z_{CPE}=\frac{1}{A{\left(j\omega \right)}^{n}}$$

where *j* is the imaginary unit (*j*^2^ = −1) and ω the angular frequency (*ω* = 2πf, f the frequency), with *A* being a constant independent of frequency [19]. *n* is an exponent index measuring the arc depression ranging between zero and unity and determines the degree of deviation from an exact semicircle.

In Equation (2), when the constant *n* = 1, we are in the case of a typical Debye behavior where the *CPE* behaves as an ideal capacitor with a value *A* = *C*, and it acts as a pure resistor in case *n* = 0 and takes the value *R* = 1/*A*. More calculation details of the parameter *n* were given in our previous work [19].

The semicircles were well-fitted based on the expressions of both $Z^{\prime }$ and $Z^{\prime \prime }$:

(3) $$\begin{array}{ll} Z^{\prime } & =\frac{R_{b}*\left(1+R_{b}*A_{b}*\omega^{n_{1}}*\cos\left(\frac{\pi }{2}n_{1}\right)\right)}{1+2*R_{b}*A_{b}*\omega^{n_{1}}*\cos\left(\frac{\pi }{2}n_{1}\right)+{\left(R_{b}*A_{b}*\omega^{n_{1}}\right)}^{2}}\\ & +\frac{R_{int}*(1+R_{int}*A_{int}*\omega^{n_{2}}*\cos\left(\frac{\pi }{2}n_{2}\right))}{1+2*R_{int}*A_{int}*\omega^{n_{2}}*\cos\left(\frac{\pi }{2}n_{2}\right)+{\left(R_{int}*A_{int}*\omega^{n_{2}}\right)}^{2}}\; \end{array}$$

(4) $$\begin{array}{ll} -Z\prime \prime & \\ =\frac{R_{b}{}^{2}*A_{b}*\omega^{n_{1}}*\sin\left(\frac{\pi }{2}n_{1}\right)}{1+2*R_{g}*A_{g}*\omega^{n_{1}}*\cos\left(\frac{\pi }{2}n_{1}\right)+{\left(R_{g}*A_{g}*\omega^{n_{1}}\right)}^{2}}\\ +\frac{R_{gb}{}^{2}*A_{gb}*\omega^{n_{2}}*\sin\left(\frac{\pi }{2}n_{2}\right)}{1+2*R_{int}*A_{int}*\omega^{n_{2}}*\cos\left(\frac{\pi }{2}n_{2}\right)+{\left(R_{int}*A_{int}*\omega^{n_{2}}\right)}^{2}} \end{array}$$

where *R_b_* and *A_b_* designed the resistive and capacitive components of the bulk region, respectively. *R_int_* and *A_int_* are the resistive and capacitive components of the interfacial impedance that correspondingly determine the space charge polarization [17]. The parameters *n*_1_ and *n*_2_ are the exponential indexes relatives to the bulk region and the interface.

**Figure 6:**
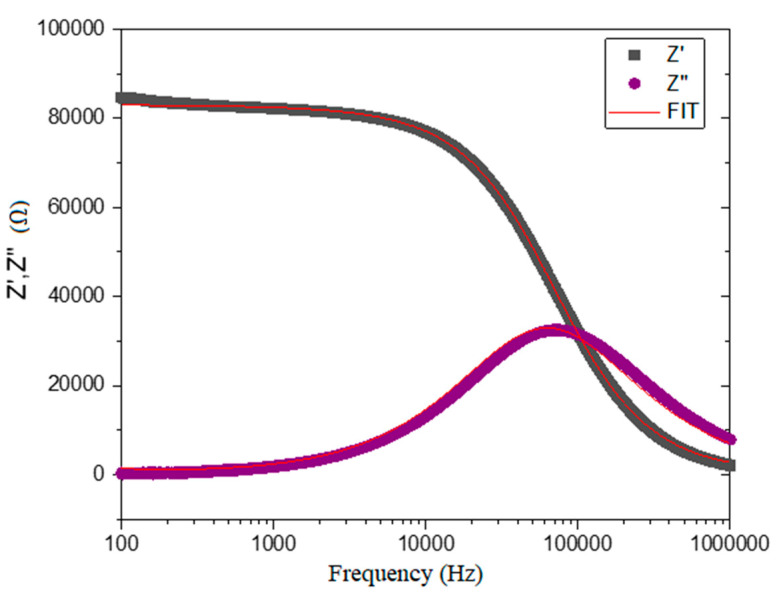
Experimental and theoretical impedance diagrams of the sample TZV5 at the temperature T = 280 °C.

A good agreement between the experimental and theoretical values is obtained for TZV5 at 280 °C (see Figure 6). This demonstrates the suitability of the equivalent circuit in well-describing the electrical properties of the glasses. The resulting parameters *A* and *n* indicate a non-ideal Debye-like behavior since the arc plot (*Z*′′ vs. *Z*′) is depressed for all the V_2_O_5_ content, and its center is below the real axis [20,21]. We attribute the deviation from the Debye profile to the formation of macroscopic dipole groups and/or nonpolar clusters [22].

**Figure 7:**
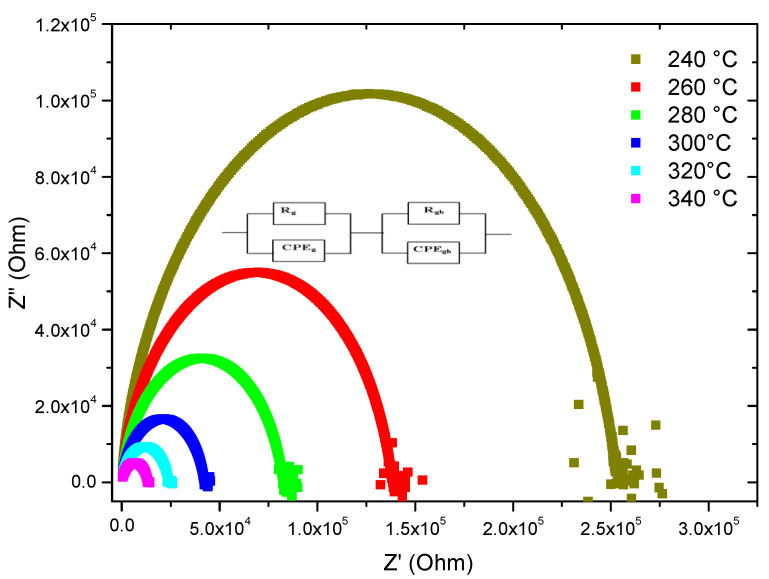
Experimental and theoretical impedance diagrams of the sample TZV5. Inset is the corresponding equivalent circuit at different temperatures.

The parameters of the equivalent circuit model are obtained based on the complex impedance Z* formula for sample TZV5 at different temperatures (see Figure 7).

The refinement results and comparisons with other works are listed in Table 1 [8,9,14]. The calculation demonstrates a gradual decrease with increasing temperature for the interfacial resistance *R_int_* and bulk resistance *R_b_*. The diminution of *R_int_* is assigned to the ionic absorption [23], which favors the ionic conductivity. On the other hand, the decrease in *R_b_* leads to an increase of the polaronic conductivity [15]. Indeed, both processes contribute to the total conductivity. Hence, the particular decrease of the radius of the semicircle in the Nyquist diagram observed for the TZV5 at 340 °C and its shift to high frequencies could be assigned to the low value of *R_b_* compared to the other samples. Thus, the conductivity is remarkably increased at this temperature. On the other hand, the higher the value of *R_int_* is, the less the hopping of the charge carriers with polaron occurs.

#### 3.2.2. Electrical Conductivity

The DC conductivity is expressed as follows:

(5) $$\sigma_{dc}=\frac{e}{SR_{b}}$$

*S* is the area, and *e* is the thickness of the sample. *R_b_* is the bulk resistance defined in the previous section.

**Figure 8:**
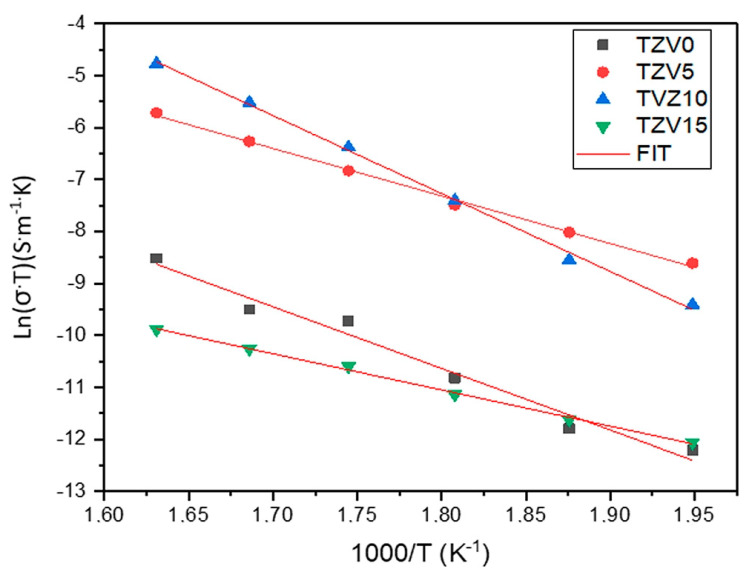
Arrhenius plots of electric dc conductivity of the TZV glasses.

Figure 8 illustrates the Arrhenius behavior of the DC conductivity:

(6) $$\sigma_{dc}T=\sigma_{0}exp(\frac{-E_{a}}{K_{B}T})$$

where *σ*_0_ is the pre-exponential factor, *E_a_* is the activation energy for conduction, *K_B_* is the Boltzmann constant, and *T* represents the absolute temperature.

*E_a_* could be calculated using the fitted curves *σ*_DC_T against reciprocal temperature (1000/T) as listed in Table 2 and compared to other glasses [18,24,25]. The highest and the lowest values of *E_a_* were founded for the TZV10 and TZV15 glasses, correspondingly.

**Figure 9:**
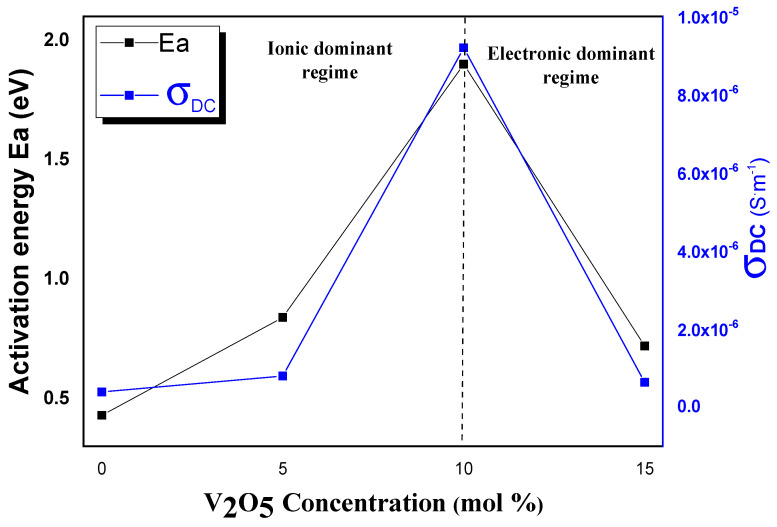
Variation of the activation energy *Ea* and the conductivity σ_DC_ at 573 K with the V_2_O_5_ content in zinc-tellurite glass samples.

The curves describing the variations of *E_a_* and *σ*_DC_ (at 300 °C) with V_2_O_5_ content show a competition between two conduction mechanisms (see Figure 9).

Indeed, two independent paths of conduction mechanisms exist so far. The first is due to the exchange interaction of V^4+^-O–V^5+^ chains, while the second corresponds to ionic conductivity described through the migration position of non-bridging oxygen along the network-former chains [27]. The electronic conduction is explained using polaron hopping between V^+4^ and V^+5^ [28]. Moreover, further addition of vanadium generates larger columbic attraction force between ions and polarons, which reduces the electronic and ionic mobility [29]. This leads to incompatible changes in bulk domain resistance and interfacial contribution, raising a challenge between the polaron and ionic conduction domination. Therefore, both electrons and holes could be associated with the local defects. Consequently, the activation energy may also include an energy to liberate the charge carrier from its position next to the defect [30].

Stambouli et al. [14] demonstrated the incorporation impact of a modified oxide in the tellurium network. Indeed, an important increase of the number of non-bridging oxygens was found through a gradual replacement of trigonal bipyramids (TeO_4_) units with trigonal pyramids (TeO_3_) through (TeO_3+1_). Thus, new units emerged in the glassy host with a high ability in switching conductivity from ionic to electronic with the V_2_O_5_ amount. The ionic conductivity dominated the conduction mechanism up to 10 mol% of V_2_O_5_., while the electronic conductivity was predominant above 10 mol %. In fact, polarons were formed from holes in the valence band, where charge carriers induce strong, localized lattice distortions forming a “small” polarons conduction [21,31]. The weak value of the activation energy observed for TZV5 could be explained by the governance of the ionic conduction, while the predominance of polaronic mechanism is assigned to the rising of the polaronic population. Hence, local structural deformation may be due to phonons and electrons transfer through hopping from lower to higher valence state. This effect is assigned to the dominance of the activation energy value associated with the predominance of electronic conduction illustrated through the formation of VO_4_ and VO_5_ structural units in the glassy network, as it was concluded from Raman analysis. Indeed, Raman spectra reveal the increase of the NBO with the V_2_O_5_ content since the intensity bands around ~210 cm^−1^ increase with a maximum for 10% mol V_2_O_5_. This could also explain the higher value of the activation energy for this sample. The co-existence of both ionic and polaronic mechanisms remains of important interest for electrochemical devices [31]. Similar behaviors related to the change of conduction process have been reported. The change of conduction mechanism has been detected for vanado-phosphate and vanado-tellurite glasses [4,32,33].

**Figure 10:**
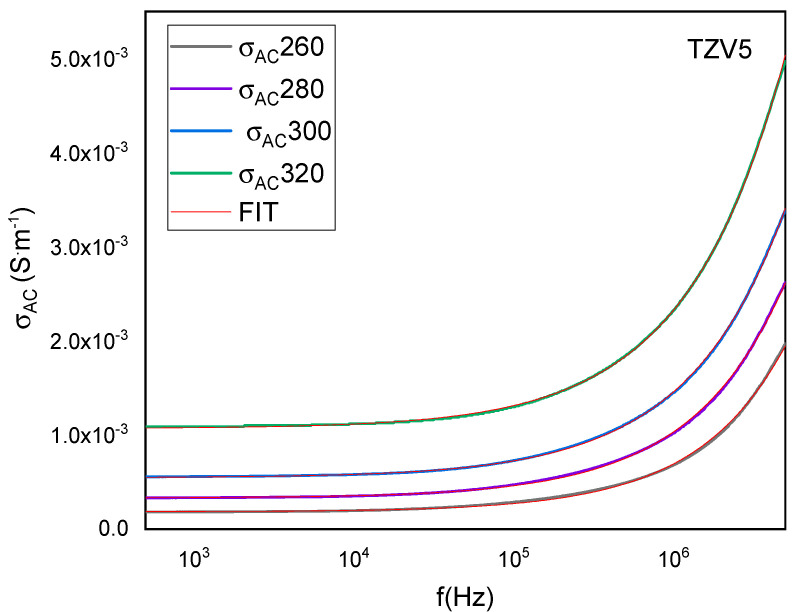
Variation versus frequency of the *AC conductivity* (*σ*_AC_) of TZV5 glass at different temperatures.

The nonlinear behavior of the isothermal σ_DC_ within the V_2_O_5_ composition could be explained by the ion–small polaron correlation process (Figure 10), where the cation–small polaron displays a similar motion that is identical to a neutral entity without affecting the conductivity [28,30].

The *AC conductivity σ*_AC_ is calculated using the expression [34]:

(7) $$\sigma_{ac}=\frac{e}{A}\frac{Z^{\prime }}{\left(Z\prime^{2}+Z{\prime \prime }^{2}\right)}$$

*σ*_DC_ remains unchanged in the range of lower frequencies, which corresponds to the DC conductivity (see Figure 10). However, in the range of high frequencies, the curves demonstrate dispersion where the slope changes to higher values with increasing temperature. This consequently changes the conductivity from the regime of frequency independence to the regime of frequency dependence, highlighting the conductivity relaxation phenomenon [35]. Hence, the necessary energy for the charge mobility at high frequencies over a short range is more important than the needed energy at low frequencies over a long range. The increase of *σ*_AC_ within the frequency sustains the hypothesis of the dominance of the hopping process in the conduction mechanism [21]. Eventually, thermally activated electrical conduction is defined as charge carriers hopping from a localized site to another with increasing temperature.

The nature and the mechanism of the conductivity dispersion in solids are generally analyzed using Jonscher’s power law [36,37]:

(8) $$\sigma =\sigma_{dc}+A\;\omega^{s}$$

where $\sigma_{\mathrm{DC}}$ is the dc conductivity, and $A\;\omega^{s}$ represents the AC conductivity. The parameter *A* determines the polarizability strength [5], and the exponent *s* (0 < *s* < 1) provides the interaction degree between the mobile charges and the lattice [38]. *A* and *s* are temperature-dependent.

Herein, “*s*” was found to slightly decrease from 0.77 to 0.71 with increasing temperature from 260 °C to 320 °C. This is due to the slight disorientation of the electric dipoles with increasing the thermal agitation [39]. Thus, the ionic conduction replaces the polaronic process, while the *AC conductivity* is explained through the correlated barrier hopping (CBH) model given by Elliot et al. [40]. In this model, the exponent *s* is written as:

(9) $$s=1-\frac{6K_{B}T}{Wm}$$

where *W_m_* is designated as the energy barrier.

#### 3.2.3. Analysis of Dielectric Constant

The real and imaginary parts *ε*′ and *ε*″ of the dielectric constant are expressed as [41,42]:

(10) $$\varepsilon \prime =-Z\prime \prime /\omega C_{0}\left(Z\prime^{2}+Z\prime \prime^{2}\right)$$

(11) $$\varepsilon \prime \prime =Z\prime /\omega C_{0}\left(Z\prime^{2}+Z\prime \prime^{2}\right)$$

where *C*_0_ is designated as the void capacitance (*C*_0_ = *ε*_0_*S*/*e*, *S* and *e* are the surface area and the sample thickness, respectively).

**Figure 11:**
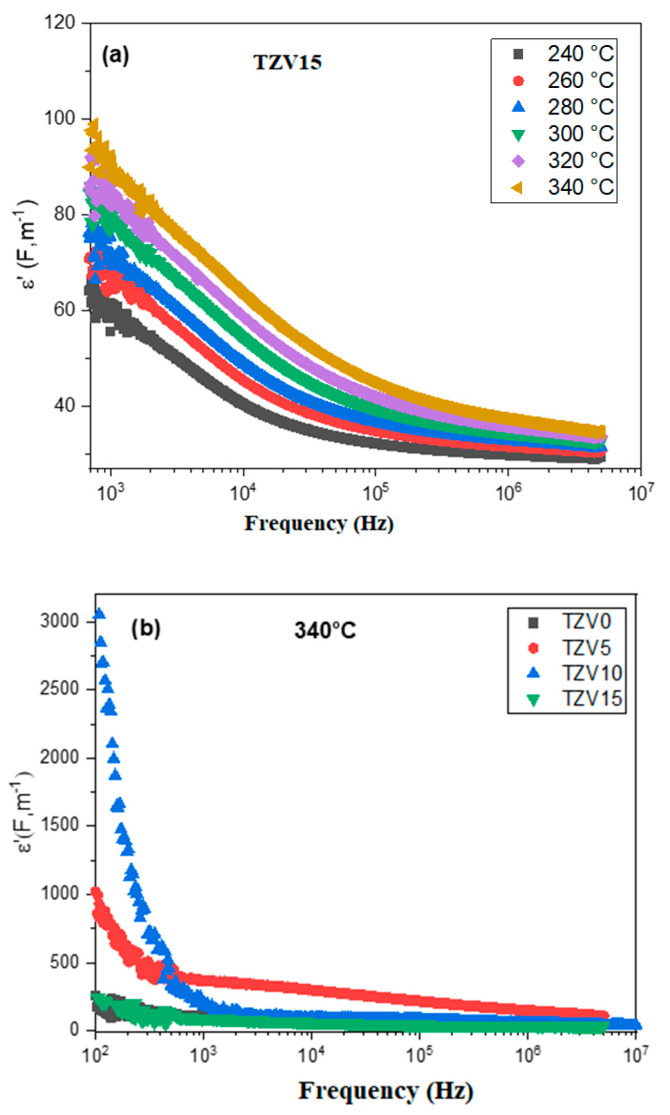
Frequency dependence of dielectric constant of (**a**) TZV15 glass sample at different temperatures and (**b**) different glass samples at T = 340 °C.

Figure 11a shows a decrease of the dielectric constant *ε*′ relative to TZV15 with the applied frequency while it increases with temperature. This is assigned to the electron hopping between two different sites [43]. The high values of *ε*′ in the range of low frequencies (Figure 11b) are related to charges accumulation at the interfaces between the electrodes and the glass, e.g., interfacial polarization [44]. The behavior of dielectric permittivity with frequency is related to the polarizability loss of some species since the hopping carriers are unable to follow the applied field [42], and their oscillations vanish with time. The relatively high values of *ε*′ relative to the TZV5 glass in the regions of low and medium frequencies make of it an appropriate material for optical devices [42].

**Figure 12:**
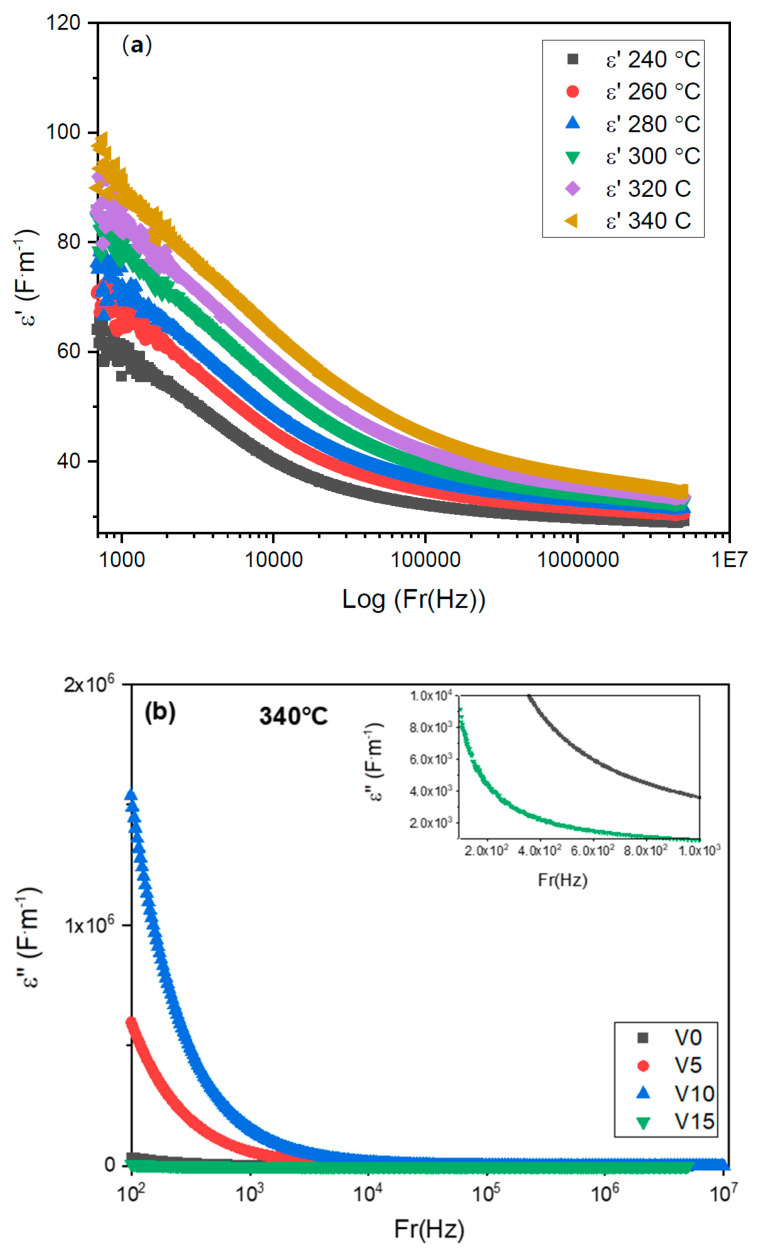
Frequency dependence of loss factor of (**a**) TZV15 glass sample at different temperatures (**b**) all TZV glass samples at T = 340 °C.

The variation of the imaginary part *ε*″ for the TZV15 glass versus the frequency at different temperatures is given by Figure 12a. Here, *ε*″ decreases gradually with the frequency up to 10^3^ Hz and then reaches a constant value *ε*″ (∞), revealing the weak dielectric loss in the range of medium and higher frequencies. Indeed, the dielectric loss usually varies with charge polarization, ionic transport, and energy diversion. Thus, the behavior of *ε*″ with the variation of the frequency could be ascribed to the fast polarization process occurring in the glass host under the applied field in addition to the dipoles generated by the electrode–electrode interface owing to the charge carriers accumulations [39,45].

Figure 12b shows the variation of the dielectric loss *ε*″ with the logarithmic frequency for all glass samples at 340 °C. *ε*″ increases with the V_2_O_5_ content till 10 mol % and then falls. The lowest value of *ε*″ is obtained for the TZV15 sample due to the formation of new phases related to vanadate (as shown from the Raman analysis), which acts as a barrier and limits the ions diffusion in the glassy host to contribute to the large reduction of the dielectric loss. It indicates the suitability of this glass for applications in photonics as electro-optic devices and nonlinear optical material.

The study of electric modulus is needed to provide deep insights into the processes of charge transport, such as relaxation phenomenon and ion dynamics. The electric modulus can be expressed as:

M* = M′(ω) + j M″(ω)(12)

where

M′(ω) = ωC_0_Z’’ (13)

M″(ω) = ωC_0_Z′ (14)

Here, C_0_ is the vacuum capacitance of the cell.

**Figure 13:**
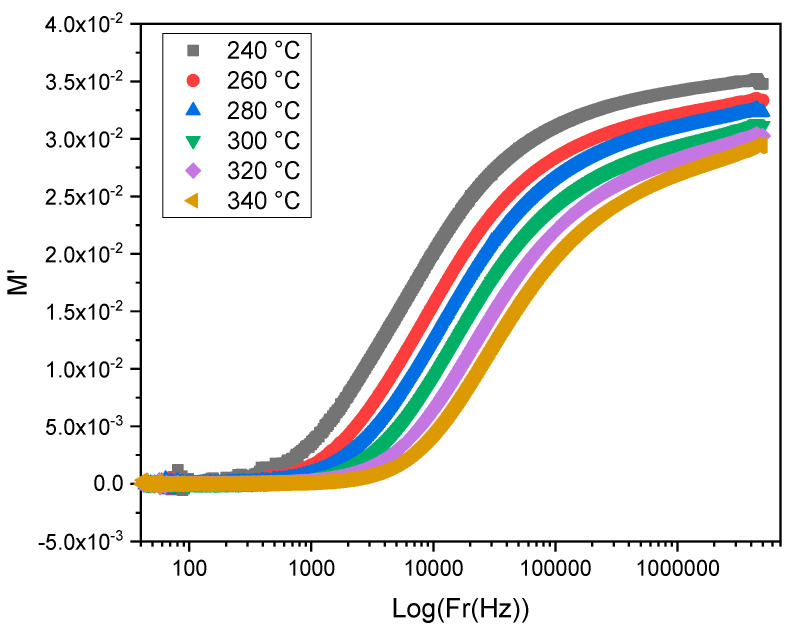
The frequency dependence of M’ for TZV15 at different temperatures.

The very small value of M′ in the region of lower frequencies indicates the suppression of the electrode polarization and the absence of long-range conduction (see Figure 13). The continuous dispersion of M′ with increasing frequency and for all the temperatures informs that the conduction process is assured through short-range mobility of charge carriers in all the glasses. This process is related to the weak restoring force in the glasses that governs the mobility of charge carriers under the influence of the applied electric field [39].

**Figure 14:**
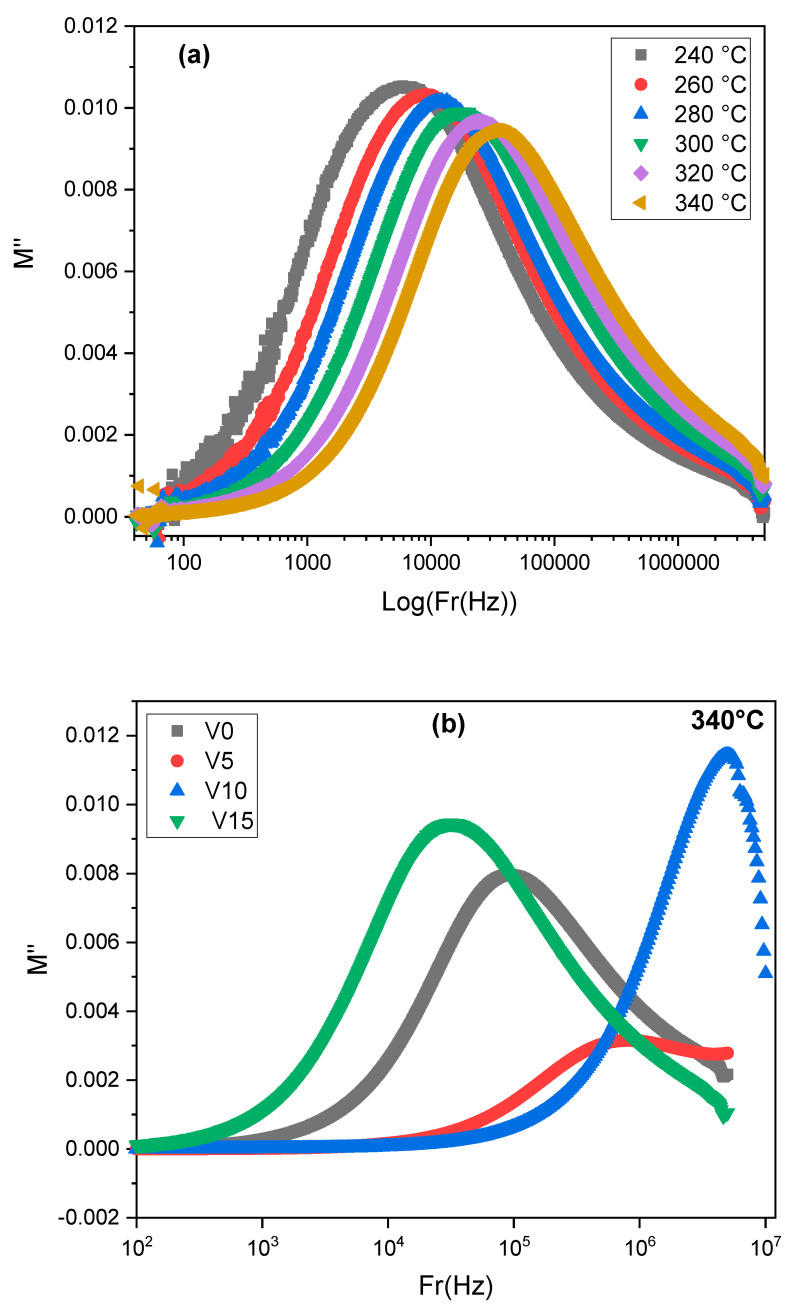
The frequency dependence of M” for (**a**) TZV15 at different temperatures (**b**) of different glass samples at T = 340 °C.

The imaginary part M″ represents a maximum peak M″_max_ within the frequency (see Figure 14a). In the frequency region below the M″ peak, the polarons drift to long distances, whereas, for the frequency range above the peak, the polarons are confined to potential wells where they are free to move. Therefore, the peak frequency represents the transition from long- to short-range mobility [46]. The asymmetric curve of M″ and its variation within the frequency at each temperature suggested a spread of the relaxation times, which can be related to a process of coupling of the individual relaxation: one site needs to relax before the other can be done [8]. On the other hand, the shift towards higher frequencies of the relaxation peak with increasing temperature indicates the dependance of the relaxation process on temperature.

Figure 14b displays the M″ curves versus frequency, at T = 340 °C, for all the V_2_O_5_ compositions. The peak position is very sensitive to the V_2_O_5_ content. The band relative to x = 10 mol % is importantly shifted to the high-frequencies range. This is related to the polarization effects of mobile ions hopping, which suggests that the relaxation time of this glass sample is lowest compared to the other glass samples. Moreover, the curve of M″(ω) confirms a large relaxation peak for the distribution, which informs again about a non-Debye model for the studied glasses.

**Figure 15:**
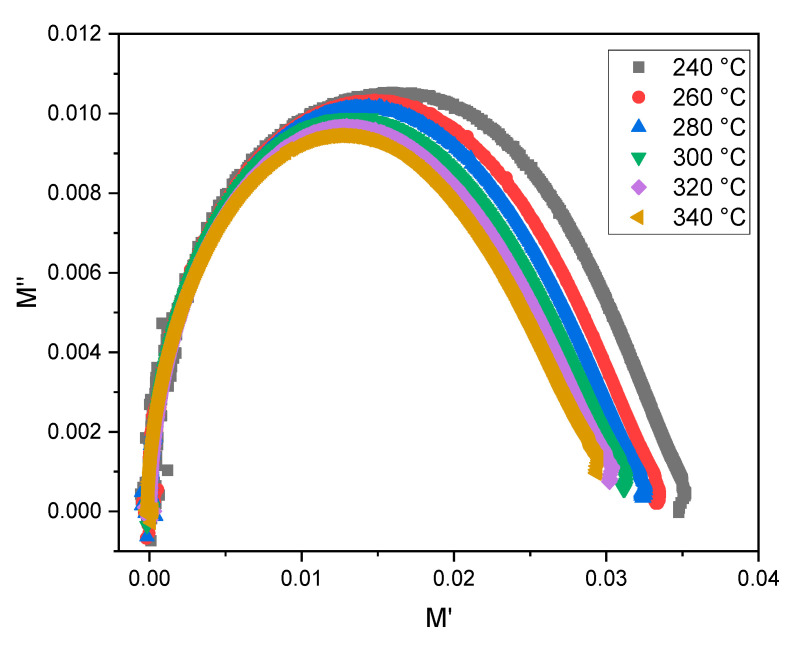
Nyquist plots for TZV15 sample at different temperatures.

Further information about the relaxation process can be obtained by analyzing the Nyquist curves (see, Figure 15). Since these curves are semicircles, and their centers are below the horizontal axis, the relationship is of a non-Debye model (single relaxation time) and corresponds to an electric relaxation and intercorrelated activation energy [24].

**Figure 16:**
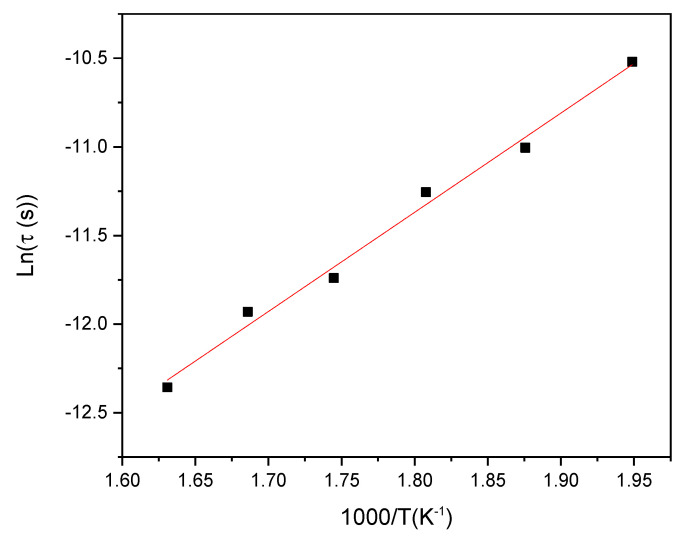
Variation of relaxation time versus 1000/T for TZV15 sample.

As shown in Figure 16, the plot of the relaxation time *τ* versus 1/*T* suggests the activation law [42]:

(15) $$\tau =\tau_{0}\;\exp\left(-\frac{E_{r}}{K_{B}T}\right)$$

From a linear fitting, the relaxation energy is estimated to *E_r_* = 0.51 eV. This value is quite different from the calculated activation energy (*E_a_* = 0.61 eV) using Equation (6). Hence, the incompatibility between the two energies indicates that the barrier height differences between the long and short distances travelled by charge carriers are not noticeable, as is highlighted by Macedo et al. [45]. Thus, the non-statistic distribution of the dipoles informs us about the arbitrary nature of conductivity. Hence, dipoles relaxation is manifested as arbitrary [9,46].

## 4. Conclusions

TeO_2_-ZnO glass systems doped with different amounts of V_2_O_5_ were prepared via the melt-quenching method. The present study clearly shows an altering of the structural, electrical, and dielectric properties of TeO_2_-ZnO glasses from addition of V_2_O_5_. The main conclusions can be mentioned as follows:

- The glass transition temperature Tg was found to increase with the V_2_O_5_ content in the glass.
- Both IR and Raman studies revealed a depolymerization of the glass network with rising the V_2_O_5_ concentration. A progressive change of the (VO_4_) groups to (VO_5_) units was shown when V_2_O_5_ composition was more than 10% by a change of the vanadium coordination due to the higher amount of NBO.
- The conductivity of the glass is assured by a mixed ionic-polaronic process with a dominance of the ionic contribution up to 10% of V_2_O_5_, whereas the polaronic component becomes the more significant above this concentration due to the exchange of polarons between V^4+^ and V^5+^.
- Variations of electric modulus and the dielectric loss with frequency and temperature exhibited dipolar relaxation effects mainly caused by the vanadate phases. In addition, the electric modulus variation shows a non-Debye dielectric dispersion.
- The decrease of the exponent “s” with temperature is consistent with the CBH process.

The good dielectric performances of the glass, such as the relatively higher dielectric constant and the low dielectric losses, are attractive for applications in optoelectronics, energy storage, and nonlinear optics.

## Figures and Tables

**Table 1:** The best-fitting values of equivalent circuit elements in Figure 7 for different temperatures.

T°	Bulk Region			Interfacial Impedance		
	*n*_1_	*A_b_* (F cm^2^ s*^n^*^−1^)	*R_b_* (Ω)	*n*_2_	*A_int_* (F cm^2^ s*^n^*^−1^)	*R_int_* (Ω)
240	0.90	6.03 × 10^−10^	2.14 × 10^6^	0.67	3.4 × 10^−6^	6.83 × 10^6^
260	0.85	9.03 × 10^−10^	1.38 × 10^6^	0.68	1 × 10^−5^	6.4 × 10^6^
280	0.85	1.91 × 10^−9^	0.83 × 10^6^	0.79	3.2 × 10^−6^	6.0 × 10^6^
300	0.85	1.03 × 10^−9^	0.42 × 10^6^	0.81	6.5 × 10^−6^	5.63 × 10^6^
320	0.84	1.34 × 10^−9^	0.24 × 10^6^	0.79	31 × 10^−5^	4.82 × 10^6^
340	0.83	1.53 × 10^−9^	0.24 × 10^6^	0.82	11 × 10^−5^	2.74 × 10^6^
LPMg [15]	0.84	1.35 × 10^−10^	0.13 × 10^6^			
NPZV10 [21]	0.86	1.774 × 10^−12^	0.136 × 10^5^	0.87	4.84 × 10^−11^	0.54 × 10^6^
PVB2.25 [18]	0.89	4.15 × 10^−11^	4.5 × 10^5^	0.87	2.7 × 10^−12^	2.3 × 10^5^

**Table 2:** Activation energies of the prepared samples and comparison with bibliography.

Host Materials	*E_a_* (eV)	References
TZV0	1.02	Present work
TZV5	0.78	Present work
TZV10	1.13	Present work
TZV15	0.62	Present work
NPZV20	0.92	[21]
90TeO_2_-10V_2_O_5_	0.428	[25]
10V_2_O_5_-Sb15-90TeO_2_	0.334	[26]
NPM5	0.91	[27]
